# Epidural anesthesia not associated with decreased 30-day surgical site infection occurrence after open colorectal surgery

**DOI:** 10.1017/ash.2026.10426

**Published:** 2026-06-25

**Authors:** Matthew Smith, Jackson Prestwood, Krista Highland, Matthew Nealeigh, Tiger Lindsay, Kayla Knuf

**Affiliations:** 1 Uniformed Services University of the Health Sciences, USA; 2 Brooke Army Medical Center, USA

## Abstract

**Objective:**

Surgical site infections (SSI) after colorectal surgery are common. Epidural anesthesia (EA) has demonstrated multiple beneficial effects for patients undergoing open colorectal surgery. However, research regarding the relationship between EA and SSI after open colorectal surgery remains limited.

**Methods:**

This retrospective study analyzed 2022–2023 data from the American College of Surgeons National Surgical Quality Improvement Project registry. Patients were included if they received a colorectal procedure without additional minor or major therapeutic procedures. Generalized additive models evaluated 30-day occurrence of any SSI (primary outcome), as well as incisional SSI and organ-space SSI (secondary outcomes). A sensitivity analysis of the primary outcome incorporated propensity score weighting to account for treatment selection.

**Results:**

Of the 6,343 patients included in the data set, 1,495 (24%) received EA and 3,391 (53%) received colorectal resection. EA was not significantly associated with any SSI (OR 1.11, 95% confidence interval (CI) 0.93–1.33, P = .26), superficial/deep incisional SSI (OR = 1.24, 95% CI 0.96–1.61, P = .11), or organ-space SSI (OR 1.00, 95% CI 0.80–1.25, P = .98). The primary findings were consistent in the sensitivity analysis (OR 0.91, 95% CI 0.77–1.08, P = .27). Patients undergoing enterostomy procedures were more likely to experience all three SSI outcomes relative to those who received colorectal resections without enterostomies.

**Conclusion:**

There was a lack of significant differences in the odds of SSI between patients who did and did not receive EA for elective open colorectal surgery.

## Introduction

Up to 18% of patients undergoing colorectal surgery, ileostomy, and other enterostomy procedures experience surgical site infections (SSIs),^1–4^ with a greater likelihood in patients undergoing open procedures.^4^ Given the regular frequency of SSI, combined with its impact on morbidity, mortality, and healthcare expenditures,^5,6^ perioperative mitigation strategies to reduce surgical infections are important. Although prior studies have identified surgical techniques to reduce SSI,^7,8^ less research has focused on anesthetic approaches.

Epidural anesthesia (EA) is associated with improved pain,^9,10^ reduced opioid consumption, improved bowel motility,^11^ shortened PACU stay,^12^ and reduced pulmonary complications^10^ after surgery. Some evidence indicates EA may also be associated with reduced rates of SSI in hip and knee surgeries.^13^ Given its benefits, EA is already a key component of several enhanced recovery after surgery (ERAS) protocols, including those for abdominal surgery,^14,15^ but its effect specifically on SSI for open abdominal surgeries is unclear.

EA may confer lower SSI probability for joint surgeries through multiple proposed mechanisms. The technique may attenuate innate immunity via reduction of cytokine production and neutrophil count.^16–18^ Additionally, EA may enhance adaptive immunity during periods of stress, particularly when excessive activation of the hypothalamic–pituitary–adrenal axis is causing systemic harm.^19^ Increased oxygen content seems to be protective in prevention of SSI^20^ and at least one historic randomized controlled trial of participants undergoing upper abdominal surgery with EA showed improved tissue oxygenation.^21^ Given the potential role of EA on SSI mitigation, evaluating real-world patient data could enable future process improvement in perioperative practices.

There is limited research on EA on SSI in abdominal surgery. A National Surgical Quality Improvement Program (NSQIP) analysis in 2018 showed no association between EA and complications in patients undergoing colorectal oncologic surgeries^22^ but did not isolate specifically for SSI outcomes. A meta-analysis in 2019 showed neuraxial anesthesia without general anesthesia was associated with reduced superficial wound infections after total knee arthroplasty, but not deep wound infections; nor was it associated with superficial or deep wound infection after total hip arthroplasty.^23^ To optimize value-based care and patient outcomes, further evaluation is needed specifically for colorectal and abdominal surgeries, to determine whether EA is associated with lower 30-day incidence of SSI in these patients. Leveraging the NSQIP database, we hypothesize that patients who receive EA will have lower SSI occurrences than those who receive general anesthesia alone.

## Methods

### Data source and record selection

This study was determined to be research not involving human participants by the Brooke Army Medical Center Institutional Review Board (BAMC IRB C.2025.030n). Data were sourced from the 2022 and 2023 American College of Surgeons NSQIP registry participant use data files. These dates were chosen to capture the most up to date ERAS and infection prevention bundles. NSQIP data is de-identified and has uniform structured fields for preoperative diagnoses, surgery information (eg, surgeon specialty, procedure code), and 30-day morbidity and mortality outcomes. To identify patients meeting inclusion criteria, classify procedures, and classify diagnoses, several tools from the Agency for Healthcare Research and Quality Healthcare Cost and Utilization Project (HCUP) were utilized.^24^ The HCUP Clinical Classification Software for Service and Procedures (CCS-SP) Current Procedural Terminology (CPT) crosswalk table was filtered for all colorectal resection or Ileostomy and other enterostomy procedures. The HCUP Surgery Flags Software for Services and Procedures were used to identify whether patients received co-occurring broad or narrow procedures based on other documented CPT codes. A single International Classification of Diseases - Version 10 (ICD-10) code indicated the presumed reason for surgery. The Clinical Classifications Software Refined (CCSR) for ICD-10-Clinical Modification Diagnoses was used to classify the two available ICD-10 diagnosis codes (Supplemental File 1). CCSR categories represented in the data were then classified by an author (MN) into five primary categories: cancer, infection, inflammation, obstruction, and other.

Next, identified CCS-SP CPT codes were used to identify the potential cohort who received an elective and open colorectal resection or ileostomy and other enterostomy procedures (N = 28,358). Patients were excluded if they did not receive general anesthesia as their primary anesthetic (n = 9,365); had contraindications to EA, presence of infection at the time of surgery, or a documented bleeding disorder (n = 809); had sepsis, septic shock, or systemic inflammatory response syndrome present before or at time of surgery (n = 1,780); were not independent, had ascites, used a ventilator, had congestive heart failure, or required dialysis (n = 1,286); had a body mass index <15 or >49.9 (n = 265), received co-occurring procedures (n = 7,745); and had more than one diagnostic category documented (n = 765). Therefore, 6,343 patients were included in the analyses.

### Outcomes

The primary outcome was a 30-day occurrence of a SSI. The secondary outcomes were 30-day occurrence of (a) incisional SSI (deep or superficial) and (b) organ-space SSI.

### Potential covariates

The primary covariate was the receipt of EA (yes/no). Additional potential covariates based on prior literature^1–5,25–27^ included the primary procedure (colorectal resection vs Ileostomy and other enterostomy procedures), age, gender, diabetes mellitus, tobacco use, chronic obstructive pulmonary disease, hypertension, steroid receipt, body mass index, American Society of Anesthesiologists (ASA) physical status classification system level (I or II vs III–V), surgical duration (minutes), length of stay (days), and diagnostic category. Approximately 19% of patients had missing race and ethnicity data, and missingness at random could not be conferred. Therefore, race and ethnicity were not included as potential covariates.

### Analytic plan

Non-parametric bivariate analyses (eg, χ^2^, distribution difference tests) assessed differences between patients who did and did not receive EA using the *compareGroups* R package.^28^ Factors that significantly varied were considered for inclusion as covariates in subsequent analyses. The outcomes were first evaluated using generalized additive models (GAMs) via the *mgcv* R package.^29^ Correction for multiplicity in the presence of multiple outcomes was not conducted, as any differences in significant covariates across models could support refined and targeted future research; and a lower significance level (eg, *P* < .025) would favor reducing Type I errors at the cost of increasing Type II errors. Therefore, the significance level remained at *P* < .05 for all three outcomes. Non-normally distributed covariates and outcomes, as well as nonlinear relationships between covariates and outcomes, can be modeled using GAMs. Continuous variables with adequate ranges (eg, age, body mass index) were not assumed to have linear relationships with the outcomes and were therefore included as smooth terms in the models. The *sjPlot* R package^30^ was used to create odds ratio (95% confidence interval (CI)) tables for the three GAM model results. The *ggeffects* R package^31^ was used to calculate marginalized means (95% CI) of significant smooth terms, which were then graphically displayed via the *ggplot2* R package.^32^ With marginalized means, non-focal continuous covariates are set to their mean and non-focal categorical covariates are marginalized over factor levels.

A sensitivity model evaluated the primary outcome (SSI) using propensity score weighting for causal inference with observational data via the *PSweight* R package.^33^ Propensity weights were constructed using all potential covariates and the overlap-weighting method. Per previous research, the overlap-weighting method was selected due to its ability to perform better than inverse probability treatment weighting in the presence of outliers, while minimizing bias in estimation.^34^ With overlap-weighting, the standardized mean difference among all weighted variables is 0. In the sensitivity analyses, records with missing data were removed in addition to those trimmed.

## Results

Of the 6,343 patients included in the data set, 1,495 (24%) received EA and 3,391 (53%) received colorectal resection. Bivariate analyses indicated patients receiving general anesthesia only versus combined general-EA significantly varied across surgery type, age, body mass index, ASA level, surgical duration, length of stay, and diagnosis category (Table 1). Therefore, these variables were included as covariates in the GAMs. The results of the GAMs are reported in Table 2. EA was not significantly associated with any SSI (OR 1.11, 95% CI 0.93–1.33, *P* = .26), superficial/deep incisional SSI (OR = 1.24, 95% CI 0.96–1.61, *P* = .11), or organ-space SSI (OR 1.00, 95% CI 0.80–1.25, *P* = .98).

**Table 1.** Overall sample descriptive and bivariate information by epidural anesthesia receipt Table 1 long description.

	Full sample (N = 6,343)	No epidural (n = 4,848)	Epidural (n = 1,495)	*P*-value
Surgery	–	–	–	<.001
Colorectal resection	3,391 (53%)	2,837 (59%)	554 (37%)	–
Ileostomy and other enterostomy procs.	2,952 (47%)	2,011 (41%)	941 (63%)	–
Age (years)	66 [56;74]	65 [55;74]	67 [58;74]	<.001
Gender	–	–	–	.75
Female	3,095 (49%)	2,375 (49%)	720 (48%)	–
Male	3,246 (51%)	2,471 (51%)	775 (52%)	–
Non-binary	2 (0.03%)	2 (0.04%)	0 (0%)	–
Diabetes mellitus	1,269 (20%)	950 (20%)	319 (21%)	.15
Tobacco use	944 (15%)	707 (15%)	237 (16%)	.24
Chronic obstructive pulmonary disease	290 (5%)	210 (4%)	80 (5%)	.11
Hypertension	3,091 (49%)	2,348 (48%)	743 (50%)	.41
Steroid receipt	481 (8%)	385 (8%)	96 (6%)	.06
Body mass index	27 [23;31]	26 [23;30]	27 [24;31]	<.001
ASA Level I or II (reference: III–V)	1,548 (24%)	1,255 (26%)	293 (20%)	<.001
Surgical duration (minutes)	235 [136;353]	222 [127;346]	272 [171;378]	<.001
Length of stay	6 [4;8]	6 [4;8]	7 [5;9]	<.001
Diagnosis category	–	–	–	<.001
Cancer	4,011 (63%)	2,869 (59%)	1,142 (76%)	–
Infection	403 (6%)	357 (7%)	46 (3%)	–
Inflammation	330 (5%)	301 (6%)	29 (2%)	–
Obstruction	312 (5%)	287 (6%)	25 (2%)	–
Other	1,287 (20%)	1,034 (21%)	253 (17%)	–
Any surgical site infection (SSI)	862 (14%)	600 (12%)	262 (18%)	<.001
Organ-space SSI	544 (9%)	378 (8%)	166 (11%)	<.001
Superficial/deep incisional SSI	320 (5%)	224 (5%)	96 (6%)	.01

Note. Values are presented as medians (interquartile ranges) and frequencies (percentages). *P*-values correspond to nonparametric bivariate analyses comparing patients who did not and did receive epidural anesthesia. Procs. = procedures.

**Table 2.** Results from the generalized additive models evaluating surgical site infection (SSI) outcomes presented as odds ratios (ORs), 95% confidence intervals (CIs), and *P*-values Table 2 long description.

	Any SSI			Superficial/deep incisional SSI			Organ-space SSI		
*Predictors*	*OR*	*CI*	*P*	*OR*	*CI*	*P*	*OR*	*CI*	*P*
(Intercept)	0.10	0.08–0.13	**<.001**	0.06	0.05–0.08	**<.001**	0.04	0.03–0.05	**<.001**
Epidural anesthesia (ref. general anesthesia only)	1.11	0.93–1.33	.26	1.24	0.96–1.61	.11	1.00	0.80–1.25	.98
ASA Level III–V (ref. I and II)	1.03	0.84–1.26	.77	1.00	0.75–1.35	.99	1.03	0.80–1.33	.82
Ileostomy/other enterostomy procs. (ref. colorectal resection)	1.25	1.01–1.55	**.04**	0.63	0.47–0.85	**.003**	1.99	1.51–2.63	**<.001**
Diagnosis category (ref. cancer)									
Infection	0.58	0.37–0.91	**.02**	0.59	0.33–1.07	.08	0.61	0.32–1.18	.14
Inflammation	0.86	0.57–1.29	.46	0.41	0.20–0.84	**.02**	1.41	0.87–2.29	.16
Obstruction	0.95	0.63–1.45	.83	0.95	0.55–1.65	.86	1.01	0.57–1.78	.98
Other	0.91	0.75–1.12	.39	0.69	0.49–0.95	**.02**	1.09	0.86–1.40	.46
*Smooth terms*									
Age	–	–	**<.001**	–	–	.13	–	–	**<.001**
Body mass index	–	–	**<.001**	–	–	**.002**	–	–	**.002**
Surgical duration	–	–	.07	–	–	**.02**	–	–	.09
Length of stay	–	–	**<.001**	–	–	**<.001**	–	–	**<.001**

Note. Bolded *P*-values indicate *P* < 0.05. Smooth terms refer to continuous covariates that were modeled to allow for nonlinear relationships between the covariate and outcome. Therefore, only *P*-values are shown as OR (95% CI) and vary across covariate values. Ref. = reference group. Procs = procedures.

**Figure 1.**
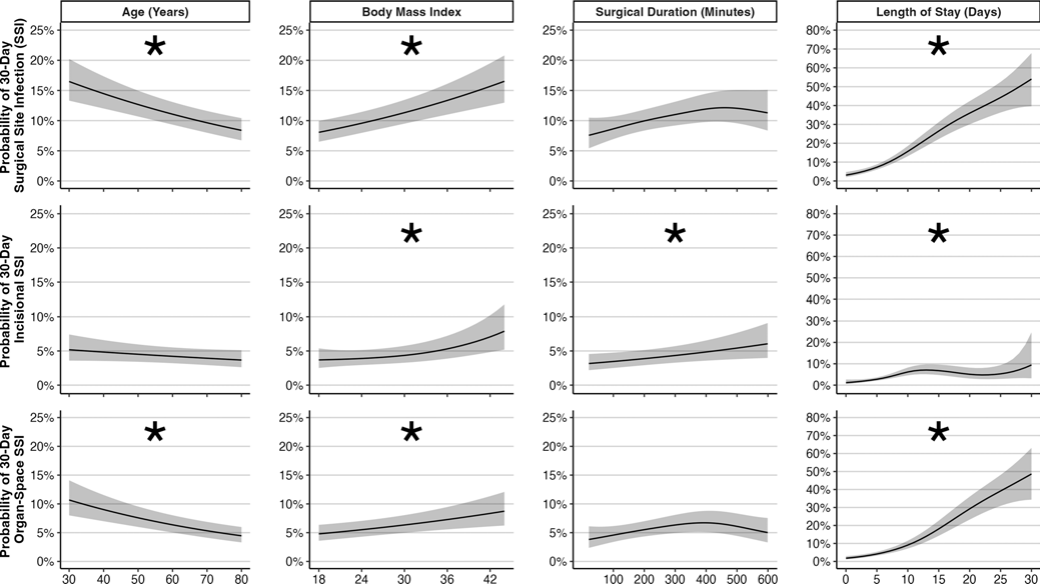
Figure 1 long description. Relationship between surgical site infection outcomes (rows) and smooth (nonlinear) covariates (columns). * *P* < .05.

In regard to other covariates, patients undergoing ileostomy and other enterostomy procedures were more likely to experience all three SSI outcomes, relative to those who received colorectal resections. Patients with a primary infection diagnosis were less likely to have any SSI, whereas patients with primary inflammation and other diagnoses were less likely to experience superficial/deep incisional SSI. As shown in Figure 1, the probability of all three SSI outcomes increased with increasing body mass index and length of stay. The probability of any SSI and organ-space SSI decreased as age increased, but the relationship between age and incisional SSI lacked significance. Lastly, the probability of incisional SSI increased with increasing surgical duration, but the relationship between any or organ-space SSI probability and age lacked significance.

In the sensitivity analysis the effective sample size after trimming and removing patients with missing data was 5,723 (25% received EA). The odds of SSI did not significantly vary between patients who did not and did receive EA (OR 0.91, 95% CI 0.77–1.08, *P* = .27).

## Discussion

In the present retrospective cohort study of NSQIP data, EA was not significantly associated with SSI in patients undergoing open colorectal, Ileostomy, and other enterostomy procedures. Overall, 14% of the sample experienced a SSI, with the majority being deep organ-space SSI. The overall SSI rate is similar to prior abdominal surgery studies.^4^ Organ-space SSI were more common (9%) than superficial and deep incisional SSI (5%). Such findings are different from previous literature, which found incisional SSIs were more common after colorectal surgeries,^35^ but slightly less common after pancreaticoduodenectomy.^36^ Taken together, the present findings suggest the benefit of EA may not extend to SSI mitigation in open abdominal colorectal procedures.

Several other covariates were associated with the three SSI outcomes, but relationships were nuanced. Colorectal resection was associated with greater likelihood of any SSI, but these patients had lower incisional SSI probability and higher organ-space SSI probability. The reason for this is unclear; these patients could have improved superficial blood flow relative to deeper flow, or they could have an increased likelihood of anastomotic-related infections.

Cancer was selected as the primary diagnosis reference category as it was the most represented in the data. Patients with a primary cancer diagnosis were more likely to have any SSI compared to those with a primary diagnosis of infection, inflammation, or other. Cancer can have direct immunomodulating effects and common cancer treatments can be immunotoxic.^37^ Therefore, there are many potential mechanisms for this finding, and further analysis would be necessary to provide clarity in that population.

Body mass index was positively associated with all three SSI outcomes, as was length of stay, on average. However, the relationship between incisional SSI and length of stay was nonlinear. Here, the probability increased within the first two weeks, then plateaued (Figure 1). Given the variation in significant covariates and direction of the relationships across SSI outcomes, it may be helpful to plan subanalyses when evaluating SSI occurrence in future studies

There are potential confounding factors associated with the limitations of this study. Compared to prior findings of decreased SSI probability after total knee arthroplasty with EA, the lack of relationship in the present study, could be attributed to discrepancies in the timing or density of neuraxial anesthesia, as well as administration of general anesthesia. The NSQIP data limits insights into specific EA administration practices, and there is likely marked heterogeneity in dosing practices, medication preferences, and patient selection for EA among providers. In orthopedic surgeries, neuraxial anesthesia can be the primary anesthetic and administered prior to surgical insult. Neuraxial anesthesia without general anesthesia requires greater block density than neuraxial anesthesia used as an adjunct for pain management *with* general anesthesia. In this study, <1% of the 28,358 patients undergoing the open and elective colorectal, ileostomy, and other enterostomy procedures did not have general anesthesia documented as the primary or adjunct anesthesia type (and were not included in analyses). Therefore, EA was presumably used as an adjunct and may not have reached maximum density until after surgery had started, unlike EA used as the primary anesthetic during orthopedic procedures. Due to the potential impact on cardiovascular function from transient sympathectomy, epidural catheters may be placed preoperatively for colorectal and abdominal surgeries but not fully dosed until the end of surgery. Furthermore, the NSQIP database does not track medications administered in EA or as part of the overall analgesia pathway. Parenteral opioid use may increase infection likelihood, and EA may alter the amount of parenteral opioids received.^38^

Medication details in the NSQIP database are nonspecific and limited to steroids, hypertension treatment, and diabetes mellitus management. The absence of comprehensive medication data (eg, anticoagulants, antiplatelets) which are critical determinants of EA eligibility, introduces potential confounding bias into the study. Furthermore, there are conditions where SSI is more likely, such as anastomotic leakage, which are not tracked in the NSQIP database. Perioperative antibiotic agent, time, or dose is not obtainable from the database, and these factors could affect the SSI rate independent of the presence of EA. Lastly, surgical techniques previously associated with reduced SSI likelihood^7,8,32^ were not included in this study and accounting for these techniques in future analyses, if data were made available, could enable a better understanding of when, if at all, EA is associated with SSI. This study evaluated a very limited subset of abdominal surgical procedures with a known elevated SSI rate, which may mask any EA benefit related to SSI, and may not be broadly relevant to other abdominal surgeries or surgeries in general.

## Conclusion

There was a lack of significant difference in SSI between patients who did and did not receive EA for open, elective colorectal, ileostomy, and other enterostomy procedures. However, given the limitations of this retrospective cohort study, we cannot definitively state that EA does not decrease SSI. Further studies would be needed to definitively answer this question. EA provides many potential benefits for patients undergoing these procedures and should continue to be considered as an important component to their perioperative management. Furthermore, there may be unidentified benefits of EA in patients who are already in a high inflammatory state.

## Supporting information

10.1017/ash.2026.10426.sm001 Smith et al. supplementary material Smith et al. supplementary material

## Supplementary material

The supplementary material for this article can be found at https://doi.org/10.1017/ash.2026.10426.

## Disclaimers

American College of Surgeons National Surgical Quality Improvement Program and the hospitals participating in the ACS NSQIP are the source of the data used herein; they have not verified and are not responsible for the statistical validity of the data analysis or the conclusions derived by the authors.

## Table 1. Long description

The table presents data on 6,343 patients, comparing those who received epidural anesthesia (EA) and those who did not. It includes variables such as surgery type, age, body mass index, ASA level, surgical duration, length of stay, and diagnosis category. The table shows that 1,495 patients (24%) received EA, and 3,391 patients (53%) underwent colorectal resection. Bivariate analyses revealed significant variations in surgery type, age, body mass index, ASA level, surgical duration, length of stay, and diagnosis category between patients receiving general anesthesia only and those receiving combined general-EA. These variables were included as covariates in Generalized Additive Models (GAMs). The results of the GAMs indicated that EA was not significantly associated with any surgical site infection (SSI), superficial/deep incisional SSI, or organ-space SSI. The table provides detailed data on these variables and their distributions across the patient sample.

Navigate back to Table 1..

## Table 2. Long description

The table presents results from generalized additive models evaluating surgical site infection (SSI) outcomes, displayed as odds ratios (ORs), 95% confidence intervals (CIs), and P-values. The table includes predictors such as epidural anesthesia, ASA level, ileostomy or other enterostomy procedures, and diagnosis category. It also lists smooth terms like age, body mass index, surgical duration, and length of stay. The table is divided into three sections: any SSI, superficial/deep incisional SSI, and organ-space SSI. Each section provides ORs, CIs, and P-values for various predictors. Notable findings include the association of ileostomy or other enterostomy procedures with superficial/deep incisional SSI and organ-space SSI, as well as the impact of diagnosis categories like infection and inflammation on SSI outcomes.

Navigate back to Table 2..

## Figure 1. Long description

The image contains nine line graphs arranged in a three-by-three grid. Each row represents a different type of surgical site infection (SSI): surgical site, incisional, and organ-space. Each column represents a different covariate: age in years, body mass index, surgical duration in minutes, and length of stay in days. The y-axis of each graph shows the probability percentage, while the x-axis varies based on the covariate. The graphs illustrate the relationship between these covariates and the probability of SSIs. Asterisks indicate statistical significance with a p-value less than 0.05. The probability of SSIs generally decreases with age, increases with body mass index and surgical duration, and significantly increases with the length of stay. All values are approximated.

Navigate back to Figure 1..
