# Supplementary material for: Epidural anesthesia not associated with decreased 30-day surgical site infection occurrence after open colorectal surgery
Source: Antimicrob Steward Healthc Epidemiol. 2026 Jun 25;6(1):e192. doi: 10.1017/ash.2026.10426 (PMC13312248; doi:10.1017/ash.2026.10426)
Supplement: Smith et al. supplementary material [file S2732494X26104264sup001.docx]

| **Supplemental File 1.** Clinical Classifications Software Refined (CCSR) for International Classification of Diseases Version 10-Clinical Modification Diagnoses and Diagnostic Categories. | |
| --- | --- |
| **CCSR Category** | **Diagnostic Category** |
| Gastrointestinal cancers - colorectal | Cancer |
| Endocrine system cancers - pancreas | Cancer |
| Gastrointestinal cancers - all other types | Cancer |
| Neoplasms of unspecified nature or uncertain behavior | Cancer |
| Malignant neuroendocrine tumors | Cancer |
| Gastrointestinal cancers - bile duct | Cancer |
| Secondary malignancies | Cancer |
| Gastrointestinal cancers - anus | Cancer |
| Cancer of other sites | Cancer |
| Gastrointestinal cancers - stomach | Cancer |
| Non-Hodgkin lymphoma | Cancer |
| Mesothelioma | Cancer |
| Sarcoma | Cancer |
| Breast cancer - all other types | Cancer |
| Female reproductive system cancers - all other types | Cancer |
| Female reproductive system cancers - endometrium | Cancer |
| Female reproductive system cancers - fallopian tube | Cancer |
| Female reproductive system cancers - ovary | Cancer |
| Gastrointestinal cancers - esophagus | Cancer |
| Gastrointestinal cancers - gallbladder | Cancer |
| Head and neck cancers - hypopharyngeal | Cancer |
| Male reproductive system cancers - prostate | Cancer |
| Neoplasm-related encounters | Cancer |
| Respiratory cancers | Cancer |
| Skin cancers - squamous cell carcinoma | Cancer |
| Gastrointestinal cancers - small intestine | Cancer |
| Bacterial infections | Infection |
| Septicemia | Infection |
| Intestinal infection | Infection |
| Osteomyelitis | Infection |
| Diverticulosis and diverticulitis | Infection |
| Peritonitis and intra-abdominal abscess | Infection |
| Gastrointestinal and biliary perforation | Infection |
| Appendicitis and other appendiceal conditions | Infection |
| Urinary tract infections | Infection |
| Fever | Infection |
| Infective arthritis | Infection |
| Parasitic, other specified and unspecified infections | Infection |
| Skin and subcutaneous tissue infections | Infection |
| Regional enteritis and ulcerative colitis | Inflammation |
| Noninfectious gastroenteritis | Inflammation |
| Endometriosis | Inflammation |
| Complication of transplanted organs or tissue | Inflammation |
| Drug induced or toxic related condition | Inflammation |
| Gastroduodenal ulcer | Inflammation |
| Internal organ injury, initial encounter | Inflammation |
| Muscle disorders | Inflammation |
| Other specified and unspecified liver disease | Inflammation |
| Other specified status | N/A |
| Personal/family history of disease | N/A |
| Diabetes mellitus, Type 2 | N/A |
| Cardiac dysrhythmias | N/A |
| Other aftercare encounter | N/A |
| Fluid and electrolyte disorders | N/A |
| Other general signs and symptoms | N/A |
| Hypotension | N/A |
| Circulatory signs and symptoms | N/A |
| Diabetes mellitus without complication | N/A |
| Acute pulmonary embolism | N/A |
| Cerebral infarction | N/A |
| External cause codes: complications of medical and surgical care | N/A |
| External cause codes: intent of injury, accidental/unintentional | N/A |
| External cause codes: other specified, classifiable and NEC | N/A |
| General sensation/perception signs and symptoms | N/A |
| Nausea and vomiting | N/A |
| Nerve and nerve root disorders | N/A |
| Neurocognitive disorders | N/A |
| Non-pressure ulcer of skin | N/A |
| Other specified and unspecified lower respiratory disease | N/A |
| Other specified inflammatory condition of skin | N/A |
| Respiratory signs and symptoms | N/A |
| Sedative-related disorders | N/A |
| Intestinal obstruction and ileus | Obstruction |
| Benign neoplasms | Other |
| Pancreatic disorders (excluding diabetes) | Other |
| Other specified and unspecified gastrointestinal disorders | Other |
| Anal and rectal conditions | Other |
| Postprocedural or postoperative digestive system complication | Other |
| Gastrointestinal hemorrhage | Other |
| Other specified and unspecified diseases of bladder and urethra | Other |
| Biliary tract disease | Other |
| Other specified and unspecified disorders of stomach and duodenum | Other |
| Other specified female genital disorders | Other |
| Abdominal pain and other digestive/abdomen signs and symptoms | Other |
| Digestive congenital anomalies | Other |
| Abdominal hernia | Other |
| Complication of other surgical or medical care, injury | Other |
| Genitourinary signs and symptoms | Other |
| Implant, device or graft related encounter | Other |
| Postprocedural or postoperative genitourinary system complication | Other |
| Acquired absence of limb or organ | Other |
| Encounter for prophylactic or other procedures | Other |
| Acute and unspecified renal failure | Other |
| Effect of foreign body entering opening | Other |
| Esophageal disorders | Other |
| Other specified male genital disorders | Other |
| Other unspecified injury | Other |
| Postprocedural or postoperative endocrine or metabolic complication | Other |
| Postprocedural or postoperative skin complication | Other |
| Peripheral and visceral vascular disease | Other |
| Aortic and peripheral arterial embolism or thrombosis | Other |
| Coagulation and hemorrhagic disorders | Other |
| Other specified diseases of veins and lymphatics | Other |
| Note: N/A indicates the CCSR category description was not likely associated with the surgery. | |
